# Prevalence of Late Onset Stress Symptomatology (LOSS) in geriatric combat veterans and its relation with dementia: A Pilot Study

**DOI:** 10.1192/j.eurpsy.2022.1728

**Published:** 2022-09-01

**Authors:** R. Barman, M. Detweiler

**Affiliations:** 1 Genesis Medical Center, Behavioral Health, Davenport, United States of America; 2 Veterans Affairs Medical Center, Psychiatry, Salem, United States of America

**Keywords:** Dementia, Late onset PTSD, LOSS, PTSD

## Abstract

**Introduction:**

Late onset stress symptomatology (LOSS) is a relatively new concept in combat veterans, which includes repeated but not intrusive thoughts about combat-related experiences, irritability, or nightmares that do not cause impairment of daily functioning.

**Objectives:**

The objectives of this study were to identify the LOSS phenomenon in geriatric combat veterans and to establish a correlation between LOSS and cognitive deficit ± major stressors.

**Methods:**

The electronic database was searched for the last 2 years from starting the study with the hypothesis that the LOSS phenomenon has been diagnosed with sleep, anxiety, trauma-related, or impulse control related disorders. Records were examined for trauma-related symptoms, excluding major symptoms of trauma-related stressors. The veterans were assessed objectively using LOSS, PCL-5 (PTSD checklist for DSM-5), social readjustment rating scales, and MOCA (Montreal Cognitive Assessment scale) for cognitive screening.

**Results:**

We reviewed 1329 patient records and identified 35 potential LOSS subjects. Four veterans were diagnosed with PTSD not otherwise specified, 2 with anxiety disorder unspecified, and 1 veteran with nightmare disorder. The majority (85%) of the veterans scored >40 in PCL-5, and only one veteran fulfilled the criteria for LOSS, who scored 67 on the LOSS scale. All the veterans scored ≤25 on MOCA with a significant deficit in recent recall.

**Conclusions:**

Our study shows new onset stress-related symptoms are strongly associated with significant cognitive deficits and higher individual stress levels. The onset of PTSD symptoms in older combat veterans might have been correlated with the onset of cognitive deficits, as suggested by several other studies.

**Disclosure:**

No significant relationships.

